# Appraising the Role of Circulating Concentrations of Micronutrients in Hypertension: A Two-sample, Multivariable Mendelian Randomization Study

**DOI:** 10.5334/gh.1367

**Published:** 2024-10-29

**Authors:** Yuting Liu, Chenggong Bao, Han Wang, Dongsheng Wei, Zhe Zhang

**Affiliations:** 1 The First Clinical College, Liaoning University of Traditional Chinese Medicine, Shenyang, Liaoning, China; 2 Key Laboratory of Ministry of Education for TCM Viscera-State Theory and Applications, Liaoning University of Traditional Chinese Medicine, Shenyang, Liaoning, China; 3 Key Research Office of the State Central Management Bureau on the Treatment of Sputum and Stasis Diseases, Affiliated Hospital of Liaoning University of Traditional Chinese Medicine, Shenyang, Liaoning, China

**Keywords:** hypertension, micronutrients, zinc, Mendelian randomization, genome-wide association studies

## Abstract

**Background:**

Hypertension poses a significant global health challenge, warranting exploration of novel preventive measures. This study aimed to investigate the role of circulating concentrations of various micronutrients in hypertension using a Mendelian randomization (MR) approach.

**Methods:**

Data on hypertension were obtained from FinnGen, comprising 55,917 cases and 162,837 controls of European ancestry. Fifteen micronutrients were evaluated and selected based on genome-wide association studies (GWAS) data. Instrumental single nucleotide polymorphisms (SNPs) were chosen according to strict criteria. Univariable Mendelian randomization (UVMR) analysis was conducted using the inverse variance weighted (IVW) method, supplemented by sensitivity analyses. Multivariate Mendelian randomization (MVMR) analysis was performed to assess interactions between micronutrients.

**Results:**

In UVMR analysis, the IVW method revealed a potential influence of copper (OR = 1.052, 95% CI: 1.006–1.099, *P* = 0.025) and zinc (OR = 1.083, 95% CI: 1.007–1.165, *P* = 0.031) on hypertension. Sensitivity analyses supported these findings. MVMR analysis confirmed a direct positive effect of zinc on hypertension (OR = 1.087, 95% CI: 1.026–1.151, *P* = 0.005), while adjusting for zinc attenuated the effect of copper on hypertension (OR = 1.026, 95% CI: 0.987–1.066, *P* = 0.193).

**Conclusion:**

Circulating zinc levels may be a potential risk factor for hypertension, while the association with other micronutrients remains inconclusive. These findings suggest that reducing zinc intake within a healthy range may help lower hypertension risk. Future research should further explore the role of zinc and nonlinear associations for a more comprehensive understanding.

## Introduction

Hypertension, as a prevalent cardiovascular disease, has exhibited a persistent rise in incidence, emerging as a crucial global public health issue (1). Statistics indicated that from 1990 to 2019, the population of hypertensive patients aged 30 to 79 doubled. Significant disparities exist among different countries and regions, with the prevalence in certain middle-income countries surpassing that of high-income countries (2). Hypertension not only predisposes individuals to serious complications such as cardiovascular disease and stroke but also heightens the risk of organ diseases such as those affecting the heart and kidneys, posing a significant threat to patients’ quality of life and health. Presently, the treatment for hypertension primarily comprises two main categories: pharmacological and non-pharmacological interventions. Pharmacological treatment commonly utilizes antihypertensive medications, such as β-blockers, ACE inhibitors, etc., to reduce blood pressure levels (3). Non-pharmacological interventions encompass lifestyle changes, including dietary control and increased physical activity. Despite the availability of multiple treatment methods, the management of hypertension continues to encounter numerous challenges, including suboptimal treatment outcomes and severe side effects. This challenge has prompted researchers to turn their attention to the potential role of micronutrients in the onset and treatment of hypertension, aiming to identify novel treatment avenues and strategies.

Micronutrients are essential nutrients for the human body, including calcium, iron, magnesium, vitamin A, and other elements. They play a vital role in the human body, encompassing cellular metabolism, nerve transmission, enzyme activity, and various other aspects. In recent years, Bastola et al. found a significant positive correlation between serum selenium levels and hypertension, but serum zinc and copper were not significantly positively correlated (4). Lewandowska et al. found that lower copper levels in early pregnancy were associated with a higher risk of hypertension. Zinc levels did not affect the risk of hypertension (5). Xiong et al. found a significant negative correlation between dietary folate, vitamin B6, and vitamin B12 and hypertension, indicating a protective effect of these nutrients against hypertension (6). However, current research primarily relies on observational studies, which are subject to numerous limitations. These limitations include challenges in establishing causality, susceptibility to recall bias and information bias, and the inability to adequately control for confounding factors. Consequently, there is an urgent requirement for more stringent research to elucidate the precise role of micronutrients in the onset and progression of hypertension.

In the current context, Mendelian randomization (MR) studies have emerged as a prominent and highly regarded method. Its uniqueness lies in the use of genetic variations such as single nucleotide polymorphisms (SNPs) as instrumental variables (IVs) to eliminate the influence of confounding factors, allowing for a more precise assessment of the effects of micronutrients on hypertension (7). However, the absence of MR studies on circulating micronutrients and hypertension to date underscores the importance of our research. Hence, we employed a two-sample multivariable Mendelian randomization (MVMR) study to examine the potential causal relationships between 15 micronutrients (copper, selenium, zinc, calcium, magnesium, potassium, iron, folate, carotenoids, vitamin A, vitamin B6, vitamin B12, vitamin C, vitamin D, and vitamin E) and hypertension. Our study aims to fill this knowledge gap and provide theoretical insights for future research on hypertension prevention and treatment.

## Methods

### Study design

Our study followed the STROBE-MR statement for reporting MR studies (8). We utilized a two-sample, MVMR method to explore the potential causal relationship between circulating micronutrient concentrations and hypertension. MR relies on three critical assumptions: (1) IV is closely associated with circulating micronutrient concentrations; (2) IV should remain unaffected by known or unknown confounding factors; (3) IV affects hypertension solely through circulating micronutrient concentrations (9). All studies included in genome-wide association studies (GWAS) have received approval from the respective review committees; hence, ethical approval and informed consent are deemed unnecessary.

### Outcome data sources

Genotypic data for hypertension were extracted from FinnGen, including 55,917 cases of hypertension and 162,837 controls. FinnGen is a large-scale public–private partnership aimed at collecting and analyzing genomic and health data from 500,000 participants in the Finnish biobank. Our study included only individuals of European ancestry to minimize any confounding effects due to ancestry.

### Selection of instrumental SNPs

Initially, we searched PubMed (accessed on April 12, 2024) to retrieve published observational studies or meta-analyses related to micronutrients and hypertension. The preliminary catalog comprises 21 micronutrients: copper, selenium, zinc, calcium, magnesium, sodium, potassium, iron, arsenic, cadmium, mercury, manganese, lead, folate, carotenoids, vitamin A, vitamin B6, vitamin B12, vitamin C, vitamin D, and vitamin E (4,6,10,11,12,13,14,15,16,17). Following this, we conducted additional searches in the GWAS catalog (https://www.ebi.ac.uk/gwas, accessed on April 17, 2024) to acquire GWAS data on circulating micronutrient concentrations in European populations. Sodium, arsenic, cadmium, mercury, manganese, and lead were excluded due to the absence of GWAS data. Ultimately, this study evaluated a total of 15 micronutrients, comprising copper, selenium, zinc, calcium, magnesium, potassium, iron, folate, carotenoids, vitamin A, vitamin B6, vitamin B12, vitamin C, vitamin D, and vitamin E. Detailed information on these micronutrients is provided in Table 1. Eligible genetic instruments were chosen based on the following criteria: first, each SNP had to have a genome-wide significant *P*-value< 5 × 10^–^6 and an *F*-statistic > 10 to satisfy the relevance assumption. Second, SNPs with linkage disequilibrium (LD; 10 MB clustering window and an *R*2 threshold of 0.001) were excluded to meet the independence assumption. Third, PhenoScanner V2 also excluded SNPs associated with the outcome or potential confounders to fulfill the exclusion restriction assumption.

**Table 1:** Detailed information of the GWAS datasets used in the present study.


EXPOSURE	GWAS ID	STUDY OR CONSORTIUM	SAMPLE SIZE	SNP	YEAR	ANCESTRY

Copper	ieu-a-1073	Evans DM et al.	2,603	2,543,646	2013	European

Selenium	ieu-a-1077	Evans DM et al.	2,603	2,543,617	2013	European

Zinc	ieu-a-1079	Evans DM et al.	2,603	2,543,610	2013	European

Calcium	ukb-b-8951	Ben Elsworth et al.	64,979	9,851,867	2018	European

Iron	ukb-b-20447	Ben Elsworth et al.	64,979	9,851,867	2018	European

Magnesium	ukb-b-7372	Ben Elsworth et al.	64,979	9,851,867	2018	European

Potassium	ukb-b-17881	Ben Elsworth et al.	64,979	9,851,867	2018	European

Carotene	ukb-b-16202	Ben Elsworth et al.	64,979	9,851,867	2018	European

Folate	ukb-b-11349	Ben Elsworth et al.	64,979	9,851,867	2018	European

Vitamin A	ukb-b-9596	Ben Elsworth et al.	460,351	9,851,867	2018	European

Vitamin B6	ukb-b-7864	Ben Elsworth et al.	64,979	9,851,867	2018	European

Vitamin B12	ukb-b-19524	Ben Elsworth et al.	64,979	9,851,867	2018	European

Vitamin C	ukb-b-19390	Ben Elsworth et al.	64,979	9,851,867	2018	European

Vitamin D	ukb-b-18593	Ben Elsworth et al.	64,979	9,851,867	2018	European

Vitamin E	ukb-b-6888	Ben Elsworth et al.	64,979	9,851,867	2018	European


GWAS, genome-wide association study.

### Univariable MR analysis

For each phenotype of micronutrients, after harmonizing exposure and outcome directions, the inverse variance weighted (IVW) method was employed as the primary MR analysis. In addition to IVW, sensitivity analyses were performed using the MR-Egger, weighted median, weighted mode, and simple mode methods. To ensure the reliability of the results, it was required that the results of the IVW method be statistically significant, and the results of the other four methods should be consistent with the IVW results in direction. The MR-Egger intercept test is employed to detect horizontal pleiotropy, where a *P* intercept > 0.05 indicates the absence of such pleiotropy. Additionally, we evaluate heterogeneity using the IVW method and MR-Egger regression, where *P* < 0.05 indicates its existence. Cochran’s *Q* statistic is employed for assessing heterogeneity.

### Multivariate MR analysis

Considering potential interactions between different micronutrients, we have performed MVMR analysis on micronutrients that have exhibited significance in univariable Mendelian randomization (UVMR) analysis. This enables a more accurate estimation of their direct effects on hypertension. In MVMR analysis, the instrumental SNPs chosen must meet the criteria previously described for UVMR selection.

All statistical analyses in this study were performed using the ‘TwoSampleMR’ and ‘MVMR’ packages in R software (version 4.1.0) for MR analysis. The causal effects of exposure on outcomes were presented using odds ratios (ORs) and 95% confidence intervals (CIs).

## Results

### Instrumental variables

Based on the three main assumptions of MR, a series of SNPs were obtained to predict the traits in the study. Among these SNPs, 6 were utilized for predicting copper, 8 were employed for predicting zinc, and the number of SNPs used for predicting other traits varied from 6 to 19 (Table S1). Furthermore, the *F*-statistics of SNPs are listed in Table S1, and they are all >10.

### UVMR analysis of the causal relationship between micronutrients and hypertension

**Figure 1:**
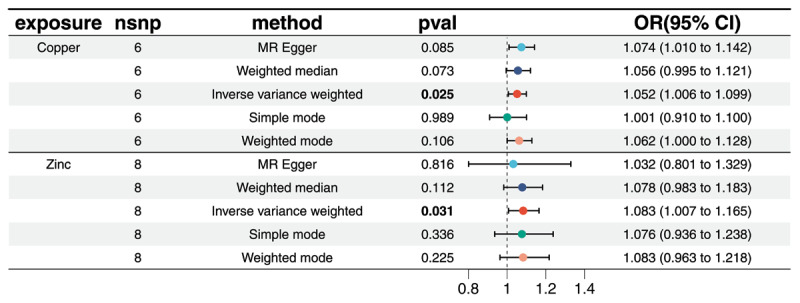
A forest plot showing associations between genetically determined levels of copper, zinc and hypertension based on IVW MR analysis. SNPs single nucleotide polymorphism, IVW inverse-variance weighted, OR odds ratio, CI confidence interval.

**Figure 2:**
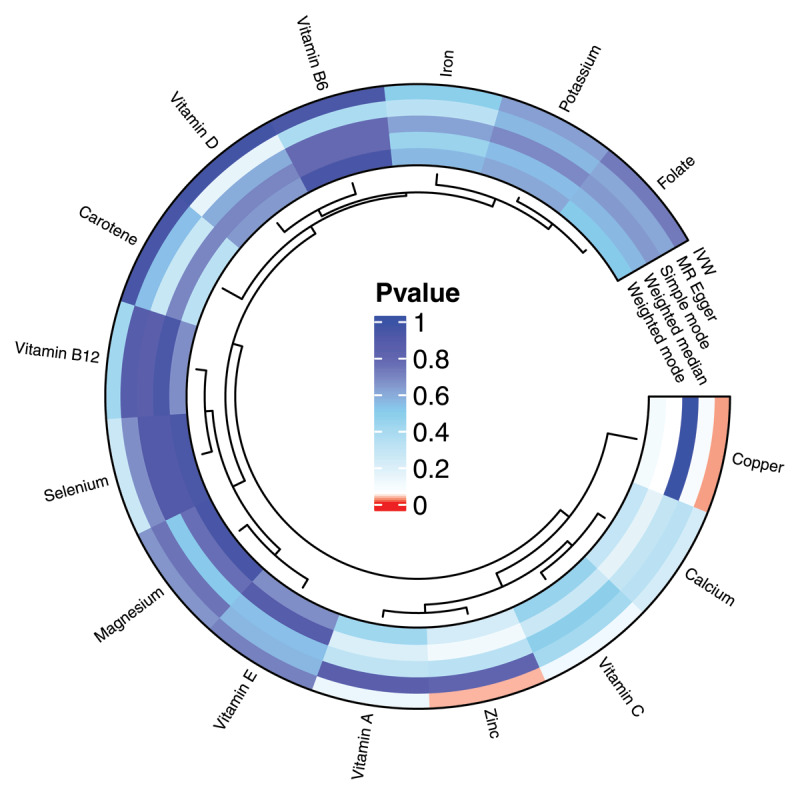
Circular heat map of suggestive genetic correlation between micronutrients and hypertension. IVW, Inverse variance weighted.

The results of the IVW method suggest a potential influence of copper on hypertension, with an OR of 1.052 (95% CI: 1.006–1.099; *P* = 0.025). Similarly, the OR for zinc is 1.083 (95% CI: 1.007–1.165; *P* = 0.031; Figure 1). Conversely, micronutrients such as selenium, calcium, magnesium, folic acid, and vitamin A have no significant impact on hypertension (all *P* > 0.05). As shown in Figure 2 and Table S2. These findings were corroborated by other MR analysis methods. Sensitivity analysis revealed heterogeneity between calcium, iron, potassium, and hypertension (Table 2). Therefore, we employed the multiplicative random-effects IVW method in this study. MR-Egger regression analyses did not detect potential horizontal pleiotropy (all*P* > 0.05), indicating that IVs do not significantly influence the outcome through pathways other than exposure (Table 2).

**Table 2:** Heterogeneity and pleiotropy analysis in MR analysis.


EXPOSURE	MR METHOD	COCHRAN Q STATISTIC	EGGER INTERCEPT	HETEROGENEITY *P*-VALUE	PLEIOTROPY *P*-VALUE

Copper	MR Egger	0.11	–0.011	0.999	0.392

Copper	IVW	1.03		0.960	

Selenium	MR Egger	3.86	0.018	0.425	0.320

Selenium	IVW	5.15		0.398	

Zinc	MR Egger	5.60	0.010	0.470	0.709

Zinc	IVW	5.75		0.569	

Folate	MR Egger	14.80	0.008	0.139	0.682

Folate	IVW	15.06		0.180	

Carotene	MR Egger	12.53	–0.010	0.484	0.471

Carotene	IVW	13.09		0.520	

Potassium	MR Egger	24.01	0.021	0.020	0.426

Potassium	IVW	25.37		0.021	

Vitamin D	MR Egger	8.45	0.040	0.672	0.136

Vitamin D	IVW	11.03		0.526	

Vitamin C	MR Egger	4.88	–0.005	0.770	0.774

Vitamin C	IVW	4.97		0.837	

Vitamin B12	MR Egger	8.48	0.013	0.205	0.606

Vitamin B12	IVW	8.90		0.260	

Iron	MR Egger	20.25	0.023	0.016	0.408

Iron	IVW	21.94		0.015	

Vitamin E	MR Egger	13.68	–0.014	0.188	0.397

Vitamin E	IVW	14.75		0.194	

Magnesium	MR Egger	11.25	–0.008	0.735	0.531

Magnesium	IVW	11.66		0.767	

Vitamin B6	MR Egger	10.23	0.013	0.805	0.350

Vitamin B6	IVW	11.16		0.800	

Calcium	MR Egger	36.73	–0.019	0.004	0.488

Calcium	IVW	37.81		0.004	

Vitamin A	MR Egger	7.25	–0.015	0.612	0.553

Vitamin A	IVW	7.63		0.665	


MR, Mendelian randomization; IVW, inverse variance weighted.

### MVMR analysis of the causal relationship between micronutrients and hypertension

**Figure 3:**
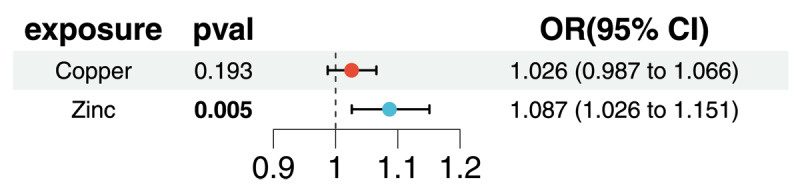
Multivariable Mendelian randomization analysis of the impact of copper, zinc on hypertension. OR odds ratio, CI confidence interval.

Due to the results of UVMR analysis showing that only copper and zinc are causally related to hypertension, the other 13 micronutrients were excluded from MVMR analysis. Ultimately, after adjusting for copper, zinc continued to demonstrate a positive direct effect on hypertension (OR = 1.087, 95% CI: 1.026–1.151, *P* = 0.005). Conversely, after adjusting for zinc, copper no longer had a direct impact on hypertension (OR = 1.026, 95% CI: 0.987–1.066, *P* = 0.193; Figure 3).

## Discussion

Hypertension is a common health issue worldwide, presenting significant challenges to global socioeconomic development. To further explore potential preventive measures, we conducted an MR study investigating the causal relationship between the levels of 15 circulating micronutrients and hypertension. Our analysis identified that circulating zinc levels may be a potential risk factor for hypertension. However, there was little evidence observed for the association of other micronutrients with the risk of hypertension.

Zinc is an essential nutrient for the human body, involved in maintaining protein structure, enzyme catalysis, and information transmission (18). However, there are multiple viewpoints and research findings regarding the relationship between zinc and hypertension. First, some studies supported the protective role of zinc in hypertension (19,20). This perspective argues that zinc deficiency can cause arteries to become stiff, fragile, and prone to inflammation, rather than flexible, which may lead to elevated blood pressure, particularly systolic blood pressure (21). However, increased zinc levels may have an inhibitory effect on inflammatory responses, helping to reduce the inflammatory response of vascular endothelial cells, thereby protecting vascular function and reducing the risk of hypertension (22,23). Additionally, zinc may lower blood pressure levels by promoting the generation of nitric oxide, which promotes vasodilation (24). Second, other studies maintain the opposite perspective, suggesting that zinc may be a risk factor for hypertension. They found that circulating zinc levels may be correlated with the occurrence and development of hypertension, indicating that higher zinc levels may be linked to an increased risk of hypertension (25,26,27). This discovery has prompted a reevaluation of the role of zinc in the pathophysiology of hypertension. This may be attributed to the role of zinc in antioxidative stress and calcium ion regulation. Zinc deficiency can result in an increase in superoxide levels, damaging endothelial structure, increasing vascular tension, and ultimately promoting the development of hypertension (28,29). Furthermore, the inhibitory effect of zinc on ATP-dependent calcium pumps may lead to an increase in vascular wall tension, further exacerbating the progression of hypertension (30,31). These perspectives offer new insights into understanding the relationship between zinc and hypertension. Apart from the above two perspectives, some studies have not found a clear association between zinc and hypertension (5). For instance, a cross-sectional study conducted by Yao et al. found no independent correlation between zinc and hypertension in the adult population in the United States (32). Bastola et al. and Darroudi et al. also reported similar findings (4,33).

Our UVMR study indicated a risk association between circulating zinc levels and hypertension. After adjusting with MVMR methods, we still observed the association between zinc and hypertension, further supporting our initial findings. This suggests that even when considering other potential influencing factors, zinc may still be one of the important risk factors for the onset of hypertension. Besides the physiological processes already mentioned, such as vascular tone regulation, antioxidative stress, and anti-inflammatory effects, some studies have found that zinc may also influence blood pressure levels through pathways such as affecting kidney function and inhibiting the sympathetic nervous system (34,35). Nevertheless, the precise mechanisms underlying the association between zinc and hypertension remain incompletely understood. The implications of these findings suggest that reducing zinc intake within a healthy range may help lower the risk of hypertension, thus informing public health strategies and clinical guidelines for hypertension prevention and management. For future research, exploring the specific pathways and molecular mechanisms through which zinc influences hypertension is critical. This could aid in the development of targeted interventions, potentially involving zinc modulation as part of personalized hypertension treatment plans. Moreover, nonlinear relationships between zinc and hypertension should be investigated, as such dynamics might better explain varying outcomes across different populations and levels of zinc intake.

Although our study did not find a clear causal relationship between other micronutrients and hypertension, there are still some possible mechanisms worthy of attention. For example, copper may affect the blood pressure regulatory system through pathways such as participating in angiogenesis and regulating vascular constriction and dilation (36). Iron, as a component of hemoglobin and myoglobin, may be related to vascular function and oxygen delivery, thereby affecting blood pressure levels (37). Magnesium is involved in multiple biochemical reactions, including neural–muscular conduction and cell signal transduction, potentially exerting a regulatory effect on blood pressure regulation (38). Furthermore, vitamin A participates in retinal generation and cell differentiation, potentially influencing vascular health and consequently affecting blood pressure regulation (11). Vitamin B6 plays a role in amino acid metabolism and neural transmission, which may affect vascular function and the nervous system, thereby influencing blood pressure (39). Despite the lack of direct evidence supporting the causal relationship between these micronutrients and hypertension in our study, future research can continue to explore their potential roles in the pathogenesis of hypertension and verify their associations with hypertension. In conclusion, our study offers some new insights into understanding the relationship between micronutrients and hypertension, while also posing numerous questions and challenges for future research to delve into.

The primary strength of this study lies in the novel application of MVMR methods to investigate the causal relationship between various circulating micronutrients and hypertension. This approach controls for confounding factors, enabling a more precise evaluation of the impact of micronutrients on hypertension and offering a fresh perspective for hypertension prevention and treatment. It is worth noting that this study has some limitations. First, the MR method assumes a linear relationship between exposure and outcome, which may not hold when nonlinear associations exist. Micronutrients may be beneficial within certain ranges, but deviations could lead to different outcomes. Our study does not account for nonlinear effects or provide specific zinc thresholds for hypertension, limiting its clinical applicability. Future research should determine optimal zinc concentrations and thresholds for intervention. Second, although MR helps to minimize confounding and reverse causality, it cannot fully eliminate bias from pleiotropy, where genetic variants affect the outcome through pathways unrelated to the exposure of interest. This may introduce some residual confounding in the results. Additionally, our study relies on data from publicly available GWAS datasets, which may lack certain individual-level details such as ethnicity, age, or sex-specific effects. These factors could influence the observed associations, thus potentially limiting the generalizability of the findings to broader populations. Finally, we only considered the effects of circulating micronutrients and did not take into account other factors that may influence hypertension, such as diet and lifestyle. These factors may have significant implications for the onset and development of hypertension, so future research should consider these factors holistically to gain a more comprehensive understanding of the pathogenesis of hypertension.

## Conclusion

In summary, our MVMR study highlights zinc’s potential as a risk factor for hypertension, while other micronutrients showed no significant association. These findings suggest that reducing zinc intake within a healthy range may help lower the risk of hypertension. Future research should delve deeper into understanding the role of zinc and explore nonlinear associations for a more comprehensive understanding.

## Data Accessibility Statement

All summary-level data necessary to conduct this MR analysis were obtained from https://gwas.mrcieu.ac.uk/.

## Additional File

The additional file for this article can be found as follows:

10.5334/gh.1367.s1 Supplementary Materials. Supplementary Tables 1 and 2.

## Abbreviations

MR, Mendelian randomized; GWAS, genome-wide association studies; SNP, single nucleotide polymorphism; UVMR, univariable Mendelian randomization; IVW, inverse variance weighting; MVMR, multivariate Mendelian randomization; OR, odds ratio; CI, confidence interval; IV, instrumental variable; LD, linkage disequilibrium.
