# Supplementary material for: Appraising the Role of Circulating Concentrations of Micronutrients in Hypertension: A Two-sample, Multivariable Mendelian Randomization Study
Source: Glob Heart. 2024 Oct 29;19(1):81. doi: 10.5334/gh.1367 (PMC11523844; doi:10.5334/gh.1367)
Supplement: Supplementary Materials. — Supplementary Tables 1 and 2. [file gh-19-1-1367-s1.pdf]

Supplementary Materials

SUPPLEMENTARY NOTES

Supplementary Table 1 | Characteristics of SNPs associated with cardiovascular disease.

Supplementary Table 2 | IVW method and sensitivity analyses using MR-Egger, Weighted median, Simple mode and Weighted mode method for the Mendelian randomization analyses of micronutrients and Hypertension.

Supplementary Table 1 | Characteristics of SNPs associated with 15 micronutrients.

Exposure	SNP	Position	EA	OA	EAF	BETA	SE	P	N	R^2	F
Calcium	rs12618785	147053856	G	C	0.399	2.73E-02	5.61E-03	1.20E-06	64979	3.63E-04	23.59
Calcium	rs4988235	136608646	A	G	0.723	3.24E-02	6.01E-03	7.40E-08	64979	4.46E-04	28.96
Calcium	rs73238581	39412384	A	C	0.347	-2.71E-02	5.70E-03	2.00E-06	64979	3.48E-04	22.64
Calcium	rs4535437	79242663	G	A	0.245	3.17E-02	6.27E-03	4.50E-07	64979	3.92E-04	25.48
Calcium	rs62347998	17168802	T	C	0.042	6.42E-02	1.38E-02	3.40E-06	64979	3.32E-04	21.58
Calcium	rs39308	116951671	A	G	0.265	2.97E-02	6.17E-03	1.50E-06	64979	3.56E-04	23.17
Calcium	rs2443773	109072593	G	T	0.506	2.72E-02	5.42E-03	5.10E-07	64979	3.88E-04	25.22
Calcium	rs1714800	3993856	C	G	0.662	2.80E-02	5.72E-03	1.00E-06	64979	3.67E-04	23.87
Calcium	rs7464794	17540356	C	T	0.499	-2.65E-02	5.39E-03	9.20E-07	64979	3.71E-04	24.08
Calcium	rs117456360	22322947	A	G	0.043	6.27E-02	1.36E-02	4.40E-06	64979	3.24E-04	21.09
Calcium	rs1219820	125445215	T	C	0.029	-7.64E-02	1.60E-02	1.70E-06	64979	3.53E-04	22.96
Calcium	rs11030416	28743834	A	G	0.042	-6.55E-02	1.36E-02	1.50E-06	64979	3.56E-04	23.16
Calcium	rs7972943	98645517	A	T	0.066	-6.76E-02	1.40E-02	1.30E-06	64979	3.60E-04	23.40
Calcium	rs753899	25947642	C	T	0.092	4.63E-02	9.35E-03	7.40E-07	64979	3.77E-04	24.51
Calcium	rs35683760	82821914	G	A	0.229	-3.10E-02	6.45E-03	1.50E-06	64979	3.56E-04	23.15
Calcium	rs8067154	77191158	A	G	0.219	-3.01E-02	6.57E-03	4.60E-06	64979	3.23E-04	21.00
Calcium	rs17712285	61755457	G	A	0.034	-7.09E-02	1.48E-02	1.70E-06	64979	3.53E-04	22.96
Calcium	rs1974821	51917735	A	G	0.152	3.57E-02	7.55E-03	2.30E-06	64979	3.44E-04	22.36
Calcium	rs8109178	12265133	T	A	0.121	3.90E-02	8.34E-03	3.00E-06	64979	3.36E-04	21.82
Calcium	rs11088797	21726149	A	G	0.114	3.96E-02	8.49E-03	3.10E-06	64979	3.35E-04	21.76
Carotene	rs1936052	112869409	T	C	0.156	-3.61E-02	7.67E-03	2.50E-06	64979	3.41E-04	22.18
Carotene	rs6660246	34362545	C	A	0.450	-2.67E-02	5.53E-03	1.40E-06	64979	3.58E-04	23.29
Carotene	rs12126792	239923493	G	A	0.012	-1.35E-01	2.80E-02	1.50E-06	64979	3.56E-04	23.14
Carotene	rs77547747	75682041	C	T	0.056	-5.58E-02	1.18E-02	2.40E-06	64979	3.42E-04	22.24
Carotene	rs6596473	138710576	C	G	0.299	2.76E-02	5.98E-03	3.70E-06	64979	3.29E-04	21.39
Carotene	rs62417408	56842667	G	A	0.038	-6.91E-02	1.47E-02	2.40E-06	64979	3.42E-04	22.24
Carotene	rs16898247	124357253	A	G	0.019	-1.07E-01	2.00E-02	9.00E-08	64979	4.40E-04	28.58
Carotene	rs13295574	139014806	A	G	0.303	-2.77E-02	5.98E-03	3.70E-06	64979	3.29E-04	21.40
Carotene	rs3829931	8125582	A	T	0.973	8.31E-02	1.78E-02	3.00E-06	64979	3.36E-04	21.81
Carotene	rs17800766	67099770	C	T	0.012	-1.22E-01	2.55E-02	1.80E-06	64979	3.50E-04	22.78
Carotene	rs2998143	135051562	G	A	0.604	-2.79E-02	5.84E-03	1.80E-06	64979	3.50E-04	22.78
Carotene	rs4771831	92025752	A	G	0.352	-2.65E-02	5.76E-03	4.20E-06	64979	3.26E-04	21.18
Carotene	rs366337	54783923	G	A	0.937	5.44E-02	1.12E-02	1.30E-06	64979	3.61E-04	23.48
Carotene	rs117731008	43746623	A	G	0.017	9.77E-02	2.12E-02	4.20E-06	64979	3.26E-04	21.19
Carotene	rs5760695	25237108	C	T	0.087	4.68E-02	1.01E-02	3.20E-06	64979	3.34E-04	21.70
Folate	rs78074774	34515489	T	C	0.045	6.01E-02	1.32E-02	4.90E-06	64979	3.21E-04	20.87
Folate	rs139588363	140739695	C	T	0.058	5.37E-02	1.17E-02	4.10E-06	64979	3.27E-04	21.23
Folate	rs3772928	115406478	C	T	0.575	-2.73E-02	5.53E-03	8.20E-07	64979	3.74E-04	24.32
Folate	rs76630415	14144445	G	T	0.212	-3.74E-02	6.70E-03	2.40E-08	64979	4.79E-04	31.11
Folate	rs2449166	3463535	T	C	0.471	2.52E-02	5.47E-03	4.00E-06	64979	3.27E-04	21.29
Folate	rs7074988	87848038	G	A	0.064	-5.13E-02	1.11E-02	4.00E-06	64979	3.27E-04	21.28
Folate	rs1502443	77060066	G	C	0.630	2.59E-02	5.64E-03	4.30E-06	64979	3.25E-04	21.11

Folate	rs16956822	7499349	A	G	0.026	-7.93E-02	1.72E-02	3.90E-06	64979	3.28E-04	21.30
Folate	rs8085166	3016615	G	A	0.677	2.78E-02	5.81E-03	1.70E-06	64979	3.52E-04	22.90
Folate	rs148031795	482310	T	C	0.015	1.04E-01	2.24E-02	3.10E-06	64979	3.35E-04	21.78
Folate	rs79975477	13683165	T	C	0.031	7.32E-02	1.56E-02	2.80E-06	64979	3.37E-04	21.92
Folate	rs76802001	31013399	A	G	0.036	-6.77E-02	1.48E-02	4.60E-06	64979	3.23E-04	21.00
Folate	rs79748722	46147495	T	C	0.028	-7.57E-02	1.65E-02	4.40E-06	64979	3.24E-04	21.07
Iron	rs17257441	117854279	T	C	0.126	-3.77E-02	8.23E-03	4.70E-06	64979	3.23E-04	20.97
Iron	rs155599	158307375	C	T	0.705	3.03E-02	5.94E-03	3.40E-07	64979	4.00E-04	26.00
Iron	rs2647238	106218428	C	T	0.587	-2.54E-02	5.49E-03	3.60E-06	64979	3.30E-04	21.46
Iron	rs148244439	107434101	T	C	0.052	5.92E-02	1.22E-02	1.30E-06	64979	3.61E-04	23.46
Iron	rs799443	44769013	A	T	0.669	-3.01E-02	5.74E-03	1.60E-07	64979	4.23E-04	27.51
Iron	rs56256289	1892529	T	C	0.187	3.26E-02	6.90E-03	2.30E-06	64979	3.43E-04	22.29
Iron	rs6463742	1424928	C	T	0.484	2.61E-02	5.56E-03	2.60E-06	64979	3.40E-04	22.12
Iron	rs9297943	87664244	G	T	0.642	2.65E-02	5.63E-03	2.40E-06	64979	3.42E-04	22.21
Iron	rs1370102	128317498	A	C	0.431	-2.52E-02	5.46E-03	3.80E-06	64979	3.28E-04	21.34
Iron	rs118189684	90693179	C	T	0.055	5.93E-02	1.21E-02	8.90E-07	64979	3.72E-04	24.15
Iron	rs114738685	107791031	T	C	0.056	5.54E-02	1.17E-02	2.40E-06	64979	3.43E-04	22.27
Iron	rs116863411	46633015	T	A	0.018	-9.47E-02	2.04E-02	3.50E-06	64979	3.31E-04	21.50
Magnesium	rs2745938	208139949	T	G	0.647	2.69E-02	5.62E-03	1.60E-06	64979	3.53E-04	22.96
Magnesium	rs116740989	104993244	A	C	0.012	1.18E-01	2.51E-02	2.80E-06	64979	3.38E-04	21.97
Magnesium	rs114575778	79596378	A	G	0.010	-1.45E-01	2.74E-02	1.20E-07	64979	4.31E-04	28.00
Magnesium	rs116028267	179052804	G	C	0.038	-6.66E-02	1.44E-02	3.90E-06	64979	3.28E-04	21.33
Magnesium	rs4535437	79242663	G	A	0.245	3.02E-02	6.23E-03	1.20E-06	64979	3.62E-04	23.50
Magnesium	rs114989460	46322293	C	T	0.018	9.58E-02	2.09E-02	4.60E-06	64979	3.23E-04	21.00
Magnesium	rs144862520	153872931	T	C	0.041	-7.03E-02	1.54E-02	4.70E-06	64979	3.22E-04	20.94
Magnesium	rs77126457	136152474	A	G	0.014	1.15E-01	2.28E-02	4.50E-07	64979	3.92E-04	25.47
Magnesium	rs76330086	110307810	T	C	0.015	-1.02E-01	2.21E-02	4.40E-06	64979	3.24E-04	21.08
Magnesium	rs7022555	137753121	T	C	0.012	-1.21E-01	2.53E-02	1.80E-06	64979	3.51E-04	22.81
Magnesium	rs1247081	30048948	T	G	0.508	2.57E-02	5.37E-03	1.70E-06	64979	3.52E-04	22.88
Magnesium	rs116979507	90779474	T	C	0.039	6.36E-02	1.39E-02	4.60E-06	64979	3.23E-04	20.99
Magnesium	rs573905	118572267	G	A	0.547	2.75E-02	5.39E-03	3.20E-07	64979	4.02E-04	26.11
Magnesium	rs147150587	28448965	A	G	0.010	1.28E-01	2.76E-02	3.40E-06	64979	3.32E-04	21.56
Magnesium	rs7339029	100899519	T	G	0.134	3.63E-02	7.92E-03	4.70E-06	64979	3.23E-04	20.96
Magnesium	rs1559583	70714102	C	T	0.785	-3.03E-02	6.54E-03	3.50E-06	64979	3.31E-04	21.51
Magnesium	rs111419911	38900761	G	A	0.310	-2.93E-02	5.88E-03	6.30E-07	64979	3.82E-04	24.81
Potassium	rs2745938	208139949	T	G	0.647	2.61E-02	5.67E-03	4.10E-06	64979	3.26E-04	21.21
Potassium	rs3772928	115406478	C	T	0.575	-2.65E-02	5.51E-03	1.50E-06	64979	3.57E-04	23.18
Potassium	rs114300683	154885392	A	G	0.012	-1.15E-01	2.49E-02	4.00E-06	64979	3.27E-04	21.26
Potassium	rs148244439	107434101	T	C	0.052	5.66E-02	1.23E-02	3.90E-06	64979	3.28E-04	21.31
Potassium	rs145857065	34461430	C	T	0.030	-8.29E-02	1.73E-02	1.60E-06	64979	3.54E-04	22.99
Potassium	rs77126457	136152474	A	G	0.014	1.05E-01	2.30E-02	4.80E-06	64979	3.22E-04	20.90
Potassium	rs35579431	9139385	T	C	0.266	2.91E-02	6.13E-03	2.00E-06	64979	3.48E-04	22.63
Potassium	rs7040926	20241351	C	T	0.065	-5.11E-02	1.11E-02	4.40E-06	64979	3.24E-04	21.08
Potassium	rs10764330	22286351	G	A	0.699	2.78E-02	5.92E-03	2.60E-06	64979	3.39E-04	22.06

Potassium	rs12412051	2601269	C	G	0.034	7.25E-02	1.49E-02	1.20E-06	64979	3.63E-04	23.63
Potassium	rs1360913	61280467	T	A	0.577	2.78E-02	5.50E-03	4.40E-07	64979	3.92E-04	25.49
Potassium	rs361294	103993635	C	A	0.691	-2.82E-02	5.93E-03	2.00E-06	64979	3.48E-04	22.63
Potassium	rs7479680	14154711	C	A	0.144	-3.81E-02	7.75E-03	9.00E-07	64979	3.71E-04	24.13
Potassium	rs12296227	101439551	T	G	0.267	2.84E-02	6.15E-03	3.80E-06	64979	3.29E-04	21.38
Potassium	rs77824658	73222641	G	A	0.090	4.38E-02	9.54E-03	4.40E-06	64979	3.24E-04	21.08
Vitamin A	rs72774943	2715467	T	C	0.061	-2.75E-03	6.01E-04	4.80E-06	460351	4.54E-05	20.90
Vitamin A	rs4131899	80320484	T	C	0.517	-1.37E-03	2.86E-04	1.70E-06	460351	4.99E-05	22.96
Vitamin A	rs72833036	24351057	G	A	0.062	3.30E-03	5.97E-04	3.10E-08	460351	6.65E-05	30.62
Vitamin A	rs10248388	94018115	C	A	0.048	3.13E-03	6.73E-04	3.40E-06	460351	4.69E-05	21.60
Vitamin A	rs4379489	1865205	G	A	0.213	1.61E-03	3.50E-04	4.30E-06	460351	4.59E-05	21.12
Vitamin A	rs1953042	90007027	C	T	0.164	1.84E-03	3.92E-04	2.60E-06	460351	4.80E-05	22.09
Vitamin A	rs3978773	102049300	A	T	0.509	-1.41E-03	2.88E-04	9.40E-07	460351	5.22E-05	24.05
Vitamin A	rs74161932	134745108	C	T	0.102	2.26E-03	4.74E-04	1.90E-06	460351	4.93E-05	22.68
Vitamin A	rs1478684	27337345	G	T	0.229	1.65E-03	3.42E-04	1.40E-06	460351	5.05E-05	23.24
Vitamin A	rs7330997	70649177	C	T	0.134	-2.03E-03	4.22E-04	1.60E-06	460351	5.00E-05	23.02
Vitamin A	rs2581667	72942835	G	A	0.858	1.95E-03	4.16E-04	2.70E-06	460351	4.79E-05	22.04
Vitamin A	rs9982066	17820437	A	C	0.489	1.46E-03	2.91E-04	5.00E-07	460351	5.48E-05	25.24
Vitamin A	rs6005584	28109329	C	G	0.089	-2.31E-03	5.03E-04	4.40E-06	460351	4.58E-05	21.07
Vitamin B12	rs10924919	247272298	T	C	0.394	-2.86E-02	5.64E-03	3.90E-07	64979	3.96E-04	25.74
Vitamin B12	rs112961770	78554861	C	G	0.023	-8.88E-02	1.84E-02	1.40E-06	64979	3.57E-04	23.23
Vitamin B12	rs67568068	160155731	C	T	0.213	-3.17E-02	6.65E-03	1.80E-06	64979	3.51E-04	22.80
Vitamin B12	rs148901823	36745068	G	A	0.081	-4.86E-02	1.01E-02	1.30E-06	64979	3.60E-04	23.41
Vitamin B12	rs12776611	71424622	A	G	0.022	-8.78E-02	1.89E-02	3.40E-06	64979	3.32E-04	21.60
Vitamin B12	rs1419875	118044136	G	T	0.201	-3.15E-02	6.83E-03	3.90E-06	64979	3.28E-04	21.29
Vitamin B12	rs61994378	106827305	C	T	0.028	9.35E-02	1.98E-02	2.30E-06	64979	3.44E-04	22.34
Vitamin B12	rs6088761	33777099	G	A	0.390	2.93E-02	6.27E-03	3.00E-06	64979	3.35E-04	21.79
Vitamin B12	rs388561	34612149	C	T	0.881	4.03E-02	8.55E-03	2.50E-06	64979	3.41E-04	22.19
Vitamin B6	rs188211816	172810049	A	G	0.029	-7.86E-02	1.63E-02	1.40E-06	64979	3.59E-04	23.32
Vitamin B6	rs155599	158307375	C	T	0.705	3.43E-02	5.98E-03	1.00E-08	64979	5.04E-04	32.76
Vitamin B6	rs3772928	115406478	C	T	0.575	-2.92E-02	5.53E-03	1.30E-07	64979	4.30E-04	27.93
Vitamin B6	rs141933624	91118956	A	G	0.021	-8.97E-02	1.93E-02	3.30E-06	64979	3.33E-04	21.66
Vitamin B6	rs183178622	103988054	T	C	0.018	-9.89E-02	2.07E-02	1.70E-06	64979	3.52E-04	22.88
Vitamin B6	rs77806858	153476869	C	T	0.070	-5.05E-02	1.06E-02	1.90E-06	64979	3.49E-04	22.66
Vitamin B6	rs12198456	120371988	T	C	0.019	9.20E-02	1.97E-02	3.10E-06	64979	3.34E-04	21.73
Vitamin B6	rs74640671	21550838	T	C	0.011	-1.27E-01	2.74E-02	3.40E-06	64979	3.32E-04	21.57
Vitamin B6	rs12412051	2601269	C	G	0.034	7.09E-02	1.50E-02	2.20E-06	64979	3.44E-04	22.39
Vitamin B6	rs361294	103993635	C	A	0.691	-2.74E-02	5.95E-03	4.30E-06	64979	3.25E-04	21.13
Vitamin B6	rs12226112	14163360	T	G	0.341	2.83E-02	5.73E-03	7.50E-07	64979	3.77E-04	24.49
Vitamin B6	rs9560457	90476979	T	C	0.404	2.55E-02	5.54E-03	4.10E-06	64979	3.26E-04	21.19
Vitamin B6	rs10138490	80883798	C	T	0.061	-5.35E-02	1.14E-02	2.80E-06	64979	3.38E-04	21.97
Vitamin B6	rs34938615	22113999	G	A	0.011	-1.22E-01	2.64E-02	3.90E-06	64979	3.28E-04	21.30
Vitamin B6	rs7205927	48911591	C	A	0.412	-2.58E-02	5.53E-03	3.20E-06	64979	3.34E-04	21.71
Vitamin B6	rs3745438	54972918	C	T	0.034	-7.13E-02	1.56E-02	4.90E-06	64979	3.21E-04	20.88

Vitamin B6	rs67450584	44202370	T	C	0.158	3.67E-02	7.46E-03	8.60E-07	64979	3.73E-04	24.23
Vitamin B6	rs7292147	17837908	C	G	0.438	-2.58E-02	5.51E-03	2.70E-06	64979	3.39E-04	22.02
Vitamin C	rs7626478	165589571	A	G	0.720	2.80E-02	6.10E-03	4.50E-06	64979	3.24E-04	21.03
Vitamin C	rs114598078	81252851	T	C	0.042	6.56E-02	1.38E-02	1.90E-06	64979	3.49E-04	22.70
Vitamin C	rs4481190	155038283	C	A	0.351	-3.06E-02	5.74E-03	9.60E-08	64979	4.38E-04	28.45
Vitamin C	rs61868302	135260228	T	C	0.061	-5.71E-02	1.18E-02	1.40E-06	64979	3.58E-04	23.26
Vitamin C	rs17482258	26949117	T	C	0.099	4.28E-02	9.26E-03	3.70E-06	64979	3.29E-04	21.39
Vitamin C	rs2018201	52340724	G	T	0.027	-8.08E-02	1.72E-02	2.50E-06	64979	3.41E-04	22.16
Vitamin C	rs9540734	66965411	A	G	0.478	-2.59E-02	5.48E-03	2.30E-06	64979	3.44E-04	22.35
Vitamin C	rs4238567	33927389	C	T	0.522	2.53E-02	5.51E-03	4.30E-06	64979	3.25E-04	21.11
Vitamin C	rs11650824	68115897	A	T	0.035	7.95E-02	1.59E-02	5.60E-07	64979	3.85E-04	25.04
Vitamin C	rs1883993	25211229	A	G	0.095	4.50E-02	9.35E-03	1.50E-06	64979	3.55E-04	23.10
Vitamin D	rs4395237	35598885	G	T	0.050	-6.01E-02	1.28E-02	2.50E-06	64979	3.41E-04	22.18
Vitamin D	rs2399949	145453826	C	T	0.185	-3.42E-02	7.06E-03	1.20E-06	64979	3.62E-04	23.52
Vitamin D	rs75713989	186632942	T	C	0.130	3.91E-02	8.17E-03	1.60E-06	64979	3.53E-04	22.98
Vitamin D	rs57038272	76259816	T	C	0.177	3.33E-02	7.24E-03	4.10E-06	64979	3.26E-04	21.21
Vitamin D	rs582962	69521978	A	G	0.684	-2.83E-02	6.08E-03	3.20E-06	64979	3.34E-04	21.69
Vitamin D	rs9328367	709090	T	A	0.510	2.62E-02	5.62E-03	3.10E-06	64979	3.34E-04	21.73
Vitamin D	rs117693112	108727403	A	G	0.041	6.82E-02	1.45E-02	2.40E-06	64979	3.42E-04	22.22
Vitamin D	rs679830	54674873	C	T	0.949	-6.02E-02	1.28E-02	2.40E-06	64979	3.42E-04	22.26
Vitamin D	rs80261862	36760624	T	C	0.098	-4.49E-02	9.27E-03	1.30E-06	64979	3.61E-04	23.45
Vitamin D	rs17301981	84986248	C	T	0.088	-4.38E-02	9.58E-03	4.90E-06	64979	3.21E-04	20.87
Vitamin D	rs74593039	44421069	C	G	0.158	3.50E-02	7.54E-03	3.40E-06	64979	3.32E-04	21.56
Vitamin D	rs61942184	103586618	C	G	0.028	8.22E-02	1.78E-02	3.70E-06	64979	3.30E-04	21.44
Vitamin D	rs35775421	103462580	A	G	0.053	-5.60E-02	1.22E-02	4.60E-06	64979	3.23E-04	20.98
Vitamin D	rs10469075	75110848	T	C	0.189	-3.25E-02	7.01E-03	3.70E-06	64979	3.30E-04	21.43
Vitamin E	rs536912	109514347	A	C	0.736	3.05E-02	6.20E-03	9.00E-07	64979	3.71E-04	24.13
Vitamin E	rs6033	169521853	G	A	0.072	-5.17E-02	1.06E-02	9.90E-07	64979	3.68E-04	23.94
Vitamin E	rs2723979	37364666	G	T	0.584	-2.66E-02	5.53E-03	1.50E-06	64979	3.57E-04	23.18
Vitamin E	rs979218	9684248	C	A	0.098	-4.30E-02	9.22E-03	3.10E-06	64979	3.35E-04	21.77
Vitamin E	rs79966958	37024435	T	C	0.013	-1.17E-01	2.45E-02	2.00E-06	64979	3.48E-04	22.61
Vitamin E	rs12421920	13249759	G	A	0.094	-4.34E-02	9.38E-03	3.70E-06	64979	3.30E-04	21.43
Vitamin E	rs111306778	97282672	A	G	0.090	-4.80E-02	9.57E-03	5.40E-07	64979	3.86E-04	25.11
Vitamin E	rs4903544	77596398	T	C	0.300	-2.95E-02	6.02E-03	9.50E-07	64979	3.70E-04	24.02
Vitamin E	rs12899673	92561644	A	G	0.333	2.69E-02	5.82E-03	3.80E-06	64979	3.29E-04	21.37
Vitamin E	rs35218694	14785162	G	A	0.034	-7.42E-02	1.53E-02	1.30E-06	64979	3.61E-04	23.46
Vitamin E	rs71385328	51353081	G	A	0.011	1.30E-01	2.62E-02	7.00E-07	64979	3.79E-04	24.61
Vitamin E	rs12165526	44361713	A	T	0.101	4.79E-02	9.18E-03	1.80E-07	64979	4.19E-04	27.24
Copper	rs2769264	151344741	G	T	0.161	3.13E-01	3.40E-02	2.63E-20	2603	3.15E-02	84.68
Copper	rs1175550	3691528	G	A	0.219	1.98E-01	3.20E-02	5.03E-10	2603	1.45E-02	38.26
Copper	rs10014072	113948790	G	A	0.672	-1.64E-01	3.40E-02	1.13E-06	2603	8.86E-03	23.25
Copper	rs12153606	84583769	T	G	0.194	-1.59E-01	3.40E-02	2.50E-06	2603	8.33E-03	21.85
Copper	rs3857536	66929048	T	C	0.552	-1.29E-01	2.80E-02	4.08E-06	2603	8.09E-03	21.21
Copper	rs12582659	76064528	C	T	0.027	1.26E+00	2.70E-01	2.86E-06	2603	8.32E-03	21.83

Selenium	rs4950779	202542202	C	T	0.034	6.73E-01	1.32E-01	3.16E-07	2603	9.89E-03	25.97
Selenium	rs10023369	63796543	A	G	0.497	-1.44E-01	2.90E-02	4.42E-07	2603	9.38E-03	24.64
Selenium	rs7700970	78411324	T	C	0.317	2.65E-01	3.70E-02	7.17E-13	2603	1.93E-02	51.26
Selenium	rs11779526	26910716	T	A	0.294	1.53E-01	3.20E-02	1.68E-06	2603	8.71E-03	22.84
Selenium	rs7163368	63571121	C	T	0.237	1.58E-01	3.40E-02	4.00E-06	2603	8.23E-03	21.58
Selenium	rs3785832	59538794	C	T	0.404	-1.46E-01	3.10E-02	1.82E-06	2603	8.45E-03	22.16
Zinc	rs10931753	154568757	C	G	0.664	-1.29E-01	2.80E-02	4.94E-06	2603	8.09E-03	21.21
Zinc	rs4333127	5930033	A	G	0.903	2.18E-01	4.70E-02	3.00E-06	2603	8.20E-03	21.50
Zinc	rs11763353	15630871	G	A	0.166	-1.92E-01	3.90E-02	6.90E-07	2603	9.23E-03	24.22
Zinc	rs1532423	86268313	G	A	0.629	-1.78E-01	2.60E-02	6.40E-12	2603	1.77E-02	46.83
Zinc	rs11232535	80928809	C	T	0.055	3.25E-01	6.50E-02	6.73E-07	2603	9.51E-03	24.98
Zinc	rs7148590	65473196	A	G	0.484	-1.40E-01	2.60E-02	1.37E-07	2603	1.10E-02	28.97
Zinc	rs10484100	86817096	G	A	0.090	-2.09E-01	4.50E-02	3.30E-06	2603	8.22E-03	21.55
Zinc	rs2120019	75334184	C	T	0.208	-2.87E-01	3.30E-02	1.55E-18	2603	2.82E-02	75.58

SNP, single-nucleotide polymorphism; EAF, effect allele frequency; EA, effect allele; OA, other allele; BETA, beta. exposure; SE, standard error; P, the Significance level of frozen shoulder; R^2 was calculated as follows: 2*BETA^2*EAF*(1-EAF). The F-statistic for each SNP was calculated as follows: F =((N-2)*(R2/ (1− R2)).

Supplementary Table 2 | IVW method and sensitivity analyses using MR-Egger, Weighted median, Simple mode and Weighted mode method for the Mendelian randomization analyses of 15 micronutrients and Hypertension.

Exposure	N snps	Method	OR	OR_lci95	OR_uci95	Pval
Copper	6	MR Egger	1.074	1.010	1.142	0.085
Copper	6	Weighted median	1.056	0.999	1.117	0.056
Copper	6	IVW	1.052	1.006	1.099	0.025
Copper	6	Simple mode	1.001	0.908	1.103	0.990
Copper	6	Weighted mode	1.062	1.002	1.127	0.099
Selenium	6	MR Egger	0.966	0.833	1.120	0.670
Selenium	6	Weighted median	1.006	0.917	1.103	0.904
Selenium	6	IVW	1.042	0.971	1.118	0.256
Selenium	6	Simple mode	0.991	0.863	1.138	0.902
Selenium	6	Weighted mode	0.996	0.901	1.100	0.936
Zinc	8	MR Egger	1.032	0.801	1.329	0.816
Zinc	8	Weighted median	1.078	0.983	1.183	0.111
Zinc	8	IVW	1.083	1.007	1.165	0.031
Zinc	8	Simple mode	1.076	0.939	1.234	0.325
Zinc	8	Weighted mode	1.083	0.963	1.218	0.227
Folate	12	MR Egger	0.796	0.341	1.856	0.609
Folate	12	Weighted median	0.881	0.574	1.351	0.560
Folate	12	IVW	0.936	0.645	1.359	0.727
Folate	12	Simple mode	0.859	0.458	1.610	0.644
Folate	12	Weighted mode	0.836	0.497	1.404	0.512
Carotene	15	MR Egger	1.228	0.647	2.332	0.541
Carotene	15	Weighted median	1.088	0.714	1.660	0.694
Carotene	15	IVW	0.990	0.736	1.331	0.947
Carotene	15	Simple mode	0.629	0.285	1.390	0.271
Carotene	15	Weighted mode	1.479	0.700	3.126	0.323
Potassium	14	MR Egger	0.658	0.168	2.572	0.558
Potassium	14	Weighted median	1.158	0.716	1.871	0.550
Potassium	14	IVW	1.124	0.697	1.811	0.632
Potassium	14	Simple mode	1.183	0.531	2.638	0.687
Potassium	14	Weighted mode	1.209	0.601	2.435	0.604
Vitamin D	13	MR Egger	0.401	0.124	1.290	0.153
Vitamin D	13	Weighted median	0.919	0.598	1.411	0.700
Vitamin D	13	IVW	1.007	0.730	1.388	0.966
Vitamin D	13	Simple mode	0.823	0.410	1.649	0.592
Vitamin D	13	Weighted mode	0.854	0.442	1.650	0.648
Vitamin C	10	MR Egger	1.496	0.604	3.703	0.410
Vitamin C	10	Weighted median	1.289	0.827	2.010	0.262
Vitamin C	10	IVW	1.317	0.930	1.866	0.121
Vitamin C	10	Simple mode	1.276	0.661	2.463	0.485
Vitamin C	10	Weighted mode	1.249	0.717	2.175	0.452
Vitamin B12	8	MR Egger	0.913	0.318	2.621	0.871

Vitamin B12	8	Weighted median	1.023	0.599	1.748	0.933
Vitamin B12	8	IVW	1.191	0.783	1.811	0.414
Vitamin B12	8	Simple mode	0.920	0.386	2.193	0.856
Vitamin B12	8	Weighted mode	0.859	0.438	1.682	0.670
Iron	11	MR Egger	0.432	0.090	2.075	0.322
Iron	11	Weighted median	0.809	0.472	1.387	0.440
Iron	11	IVW	0.829	0.487	1.410	0.489
Iron	11	Simple mode	0.783	0.307	1.994	0.619
Iron	11	Weighted mode	0.765	0.323	1.810	0.556
Vitamin E	12	MR Egger	1.228	0.623	2.423	0.566
Vitamin E	12	Weighted median	1.032	0.673	1.585	0.884
Vitamin E	12	IVW	0.940	0.677	1.306	0.713
Vitamin E	12	Simple mode	1.237	0.638	2.400	0.542
Vitamin E	12	Weighted mode	1.106	0.701	1.744	0.674
Magnesium	17	MR Egger	1.102	0.611	1.990	0.751
Magnesium	17	Weighted median	0.942	0.635	1.396	0.765
Magnesium	17	IVW	0.933	0.691	1.261	0.654
Magnesium	17	Simple mode	0.792	0.399	1.575	0.516
Magnesium	17	Weighted mode	0.979	0.503	1.903	0.950
Vitamin B6	17	MR Egger	0.738	0.383	1.423	0.379
Vitamin B6	17	Weighted median	1.054	0.715	1.553	0.792
Vitamin B6	17	IVW	0.987	0.742	1.314	0.931
Vitamin B6	17	Simple mode	1.104	0.561	2.174	0.779
Vitamin B6	17	Weighted mode	1.025	0.541	1.940	0.941
Calcium	19	MR Egger	2.229	0.461	10.778	0.333
Calcium	19	Weighted median	1.372	0.884	2.129	0.159
Calcium	19	IVW	1.286	0.858	1.927	0.224
Calcium	19	Simple mode	1.587	0.690	3.647	0.291
Calcium	19	Weighted mode	1.551	0.707	3.400	0.288
Vitamin A	11	MR Egger	1.11E+01	1.27E-10	9.72E+11	0.856
Vitamin A	11	Weighted median	2.70E-03	3.30E-07	2.21E+01	0.198
Vitamin A	11	IVW	5.41E-03	5.84E-06	5.01E+00	0.134
Vitamin A	11	Simple mode	2.74E-04	9.09E-11	8.28E+02	0.307
Vitamin A	11	Weighted mode	1.90E-03	8.06E-10	4.48E+03	0.422

OR odds ratio, CI confidence interval, SNP single nucleotide polymorphism, IVW inverse-variance weighted.
